# Modified geriatric nutritional risk index: a high-sensitivity marker with L-shaped association for sarcopenia in hospitalized older adults

**DOI:** 10.3389/fnut.2025.1686537

**Published:** 2025-11-04

**Authors:** Hua Wei, Qin Huang, Ming Liu

**Affiliations:** The Integrated Medical and Elderly Care Center, Chengdu Integrated TCM and Western Medicine Hospital, Chengdu, Sichuan, China

**Keywords:** aging, nutrition, sarcopenia, inflammation, modified geriatric nutrition risk index

## Abstract

**Background:**

Screening for sarcopenia in older inpatients is currently inadequate, primarily because of the lack of consideration of the interaction between inflammation and nutrition. This study aimed to assess the efficacy of a novel modified Geriatric Nutritional Risk Index (mGNRI), which incorporates C-reactive protein (CRP) levels and weight changes, in predicting sarcopenia compared to traditional indices (geriatric nutritional risk index, GNRI/nutritional risk index, NRI).

**Methods:**

In this cross-sectional study, we evaluated 153 hospitalized older patients (mean age, 80.2 ± 9.1 years) using comprehensive assessments. Sarcopenia was diagnosed based on the Asian Working Group for Sarcopenia (AWGS) criteria, which include muscle mass and strength/function. We analyzed the associations using restricted cubic splines and multivariable logistic regression and compared the diagnostic performance using receiver operating characteristic (ROC) analysis.

**Results:**

The prevalence of sarcopenia was 24.2% (37/153). The mGNRI was significantly lower in the sarcopenia group compared to the non-sarcopenia group (48.1 ± 11.3 vs. 56.8 ± 12.8, *p* < 0.001). The mGNRI demonstrated an L-shaped relationship with an inflection point at 55.48 (*p* for nonlinear = 0.012). Below this threshold, each unit increase in mGNRI was associated with a 16.8% reduction in the odds of sarcopenia (OR = 0.832, 95% confidence interval CI: 0.741–0.934), whereas above this point, no significant association was observed (*p* = 0.504). In contrast, the GNRI or NRI ratio showed a linear protective effect (per unit increase, OR = 0.91, *p* < 0.001). An mGNRI < 55 indicated an 8.4-fold increased risk (OR = 8.40, 95% CI: 2.69–26.20), whereas GNRI<98 or NRI < 99 indicated a 6.93-fold risk (95% CI: 2.57–18.69). Diagnostic Power: The mGNRI at a cut-off of 55 yielded a sensitivity of 80.4% and the area under the curve (AUC) of 0.752. For a GNRI<98, the balanced accuracy was characterized by a sensitivity of 75.6% and specificity of 63.8%.

**Conclusion:**

The mGNRI serves as a practical and inflammation-sensitive tool for screening for sarcopenia in older inpatients. Its L-shaped association highlights a critical intervention threshold (mGNRI<55), demonstrating superior sensitivity compared to the linear indices (GNRI, NRI). Incorporating this tool into geriatric assessments may facilitate targeted interventions to address nutritional and inflammatory needs.

## 1 Introduction

Sarcopenia is a progressive systemic disorder that affects the skeletal muscles and is characterized by a rapid decline in both muscle mass and functionality (1). Muscle atrophy is estimated to affect approximately 10–16% of the older population worldwide (2). Specifically, the prevalence of muscle atrophy in individuals aged 60–70 years ranges from 5 to 13%, whereas in those aged > 80 years, it can reach as high as 11–50% (3). In Asia, its prevalence among the older population has been reported to be between 5.5 and 25.7% (4). In China, studies indicate that 12.9 and 11.2% of community-dwelling older men and women, respectively, are affected by this condition (5). Recognized as an independent disease, muscle atrophy was assigned an International Classification of Diseases (ICD-10) in 2016 (6). This condition is often associated with an increased risk of falls and fractures, a decline in the ability to perform activities of daily living (ADLs), and loss of independence. Such adverse outcomes frequently lead to disabilities in older adults (7, 8), thereby increasing the burden on families and society.

The onset and progression of sarcopenia are closely associated with systemic inflammation and metabolic disorders. CRP, an acute-phase reactant, is a key marker for assessing the inflammatory status of the body, while serum albumin serves as a primary indicator of nutritional reserves, with lower levels indicating protein-energy malnutrition (PEM). Previous studies have shown that both CRP and serum albumin play roles in sarcopenia development (9–11). However, relying on a single indicator can be problematic because of the various factors that can influence it, complicating the independent evaluation of sarcopenia. The use of nutritional composite indicators can help mitigate this issue. The mGNRI, GNRI, and NRI are composite measures that incorporate the elements of inflammation, nutrition, and body weight. Among these, the GNRI, introduced by Bouillanne et al. in 2005 (12), is a nutritional assessment tool specifically designed for older adults and is notable for its innovative combination of serum albumin and weight deviation. The incorporation of inflammatory components into nutritional assessments represents a significant advancement in this field. While both the NRI and its adaptation to the older adults (the GNRI) provide a foundational framework, they do not sufficiently address the pathways of malnutrition influenced by inflammation. The modified GNRI (mGNRI) addresses this gap by substituting CRP for albumin. The mGNRI, GNRI, and NRI are currently recognized as prognostic factors for various chronic diseases, including cardiovascular and cerebrovascular diseases (13–15), chronic kidney disease (16), and cancer (17, 18). In contrast to other nutritional indicators, such as the Subjective Global Assessment (SGA), Mini Nutritional Assessment (MNA), and Malnutrition Universal Screening Tool (MUST), which require interviews and assessments by trained professionals, these composite indicators are clinically practical because of their usability.

Chronic low-grade inflammation accelerates muscle loss in sarcopenia, and the dual-dimensional design of the mGNRI offers theoretical advantages. However, evidence validating its diagnostic utility and mechanistic pathways remains limited. Therefore, this study aimed to investigate the relationship between mGNRI, GNRI, NRI, and sarcopenia in hospitalized older patients and to compare the performance of mGNRI, GNRI, and NRI in predicting sarcopenia.

## 2 Materials and methods

### 2.1 Study design and patients

This cross-sectional study was reviewed and approved by the Ethics Committee of the Chengdu First People’s Hospital (Grant No. 2024-YNYJ-014) to ensure adherence to ethical guidelines.

**Figure 1:**
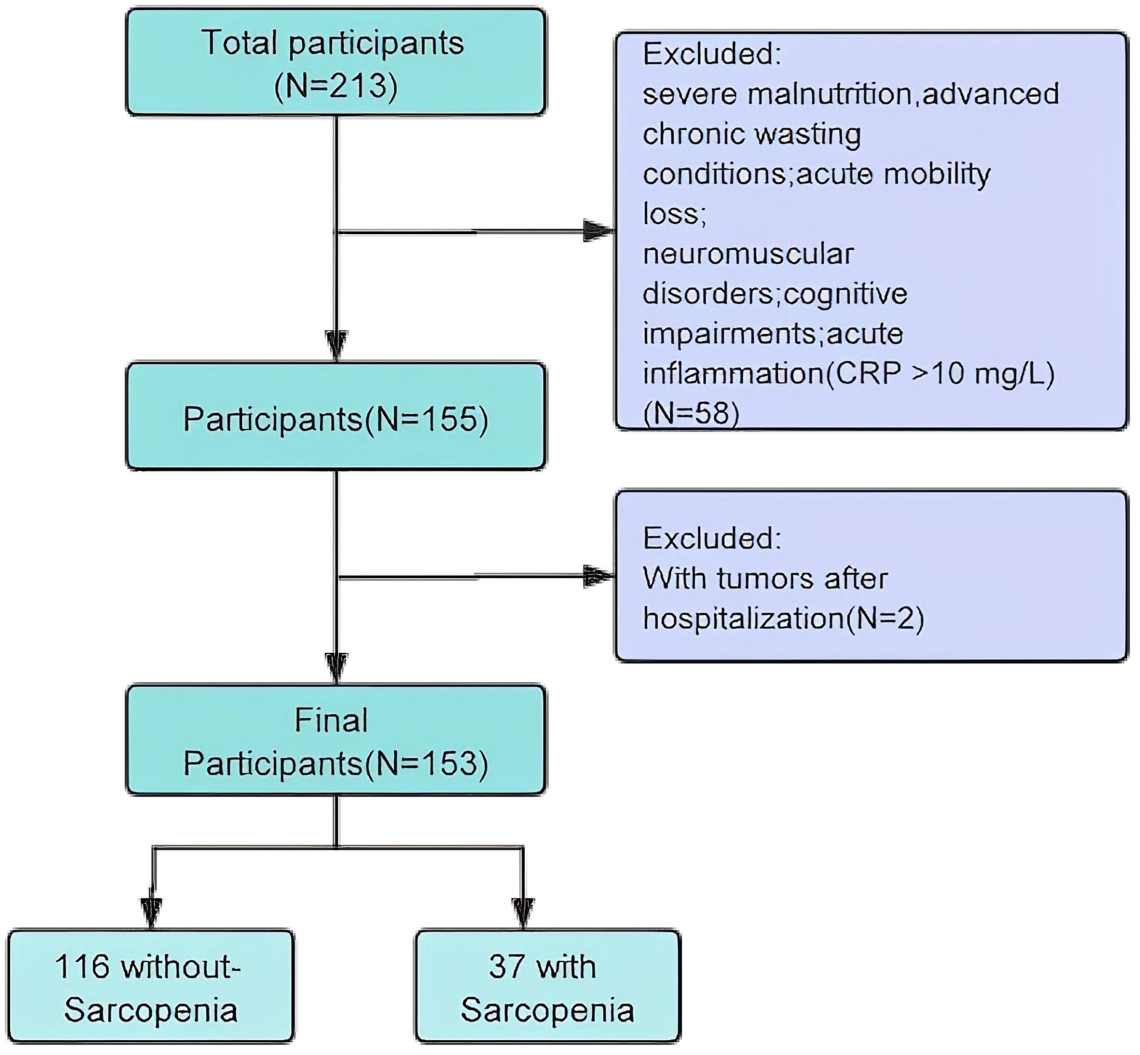
Flow chart of participant selection.

This study focused on older inpatients from the Integrated Medical and Elderly Care Center of Chengdu First People’s Hospital. Data were collected from December 2023 to May 2025, involving 153 eligible participants recruited through convenience sampling. The inclusion criteria were as follows: (1) age ≥ 65 years, (2) voluntary participation with written informed consent, (3) physical capability to perform the sarcopenia test independently, and (4) availability of complete clinical data. The exclusion criteria were as follows: (1) severe malnutrition; (2) advanced chronic wasting conditions, such as late-stage malignancies, severe chronic kidney disease (the estimated glomerular filtration rate eGFR < 30), severe diabetes, or other diseases or physiological conditions that could affect muscle mass; (3) acute mobility loss, particularly in those with fractures that limit movement, especially post-hip fractures; (4) patients diagnosed with neuromuscular disorders, such as Parkinson’s disease; (5) cognitive impairment; (6) acute inflammation (CRP > 10 mg/L) (Figure 1).

### 2.2 Data collection

#### 2.2.1 Sarcopenia diagnosis

Older inpatients who met the diagnostic criteria for sarcopenia were selected based on the AWGS: 2019 Consensus Update on Sarcopenia Diagnosis and Treatment. The diagnostic criteria were as follows: (1) muscle mass in the limbs: Bioelectrical Impedance Analysis (BIA): Appendicular Skeletal Muscle Mass (ASM) / height^2^ (kg/m^2^): for men, <7.0 kg/m^2^; for women, <5.7 kg/m^2^; (2) muscle strength: grip strength (men <28 kg, women <18 kg); (3) a walking speed of <1.0 m/s over 6 m or a time of ≥12 s for five sit-to-stand transitions. A diagnosis of sarcopenia can be made if criterion (1) is met along with any of the criteria in (2) or (3).

#### 2.2.2 Patient data

General information were collected from electronic medical records, including sex, age, height, weight, smoking history, drinking history, and comorbidities such as chronic obstructive pulmonary disease (COPD), diabetes mellitus (DM), Cavitary Cerebral Infarction (CCI), and cardiovascular disease (CVD). Grip strength and 6 m walking speed were also measured. Appendicular skeletal muscle mass (ASM) was evaluated using BIA. Routine blood and biochemical indices were obtained from fasting venous blood within 24 h after admission and sent for biochemical laboratory detection, including hemoglobin (HGB), Alanine Aminotransferase (ALT), Aspartate Aminotransferase (AST), Albumin, Total Bilirubin, Creatinine, triglycerides, CRP, and (eGFR). All data were securely stored in an electronic database, ensuring the preservation and confidentiality of the information.

### 2.3 Nutrition indicators

The calculation methods used for these three nutritional composite indicators are listed in Table 1. These methods are based on previous studies (12). The method for calculating ideal body weight was determined based on the participant’s height and body mass index (BMI) of 22 kg/m^2^.

**Table 1:** Calculation methods of combination in each nutrition indicator.

Indicators	Calculation formula
GNRI	1.489 × albumin (g/L) + 41.7 × current weight (kg)/ ideal body weight (IBW) (kg)
mGNRI	14.89/CRP (mg/L) + 41.7 × current weight (kg)/ IBW (kg)
NRI	1.519 × albumin (g/L) + 41.7 × current weight (kg)/ IBW (kg)

GNRI, geriatric nutrition risk index; mGNRI, modified geriatric nutrition risk index; NRI, nutritional risk index. If current weight was greater than IBW, current/IBW was regarded as 1. Ideal body weight is calculated using the formula based on body mass index: IBW (kg) = 22 × [height (m)]^2^.

### 2.4 Statistical analysis

Normally distributed continuous variables were reported as mean ± standard deviation (SD), while skewed continuous variables were expressed as medians with interquartile ranges (IQR). Categorical variables were presented as frequencies and percentages (%). Comparisons of continuous variables between groups were performed using either the independent samples Student’s *t*-test or the Mann–Whitney U test, based on the normality of the distribution. Categorical data were analyzed using the chi-squared test, as appropriate.

The effects of the mGNRI, GNRI, and NRI on sarcopenia were evaluated using binary logistic regression models, which reported odds ratios (OR) and 95% CI while controlling for key covariates. The mGNRI, GNRI, and NRI were treated as continuous variables with increments of one unit. Confounding factors were selected based on clinical judgment and included all covariates that were statistically significant in univariate analysis.

To assess multicollinearity among the selected covariates, we used the variance inflation factor (VIF) method, where a VIF value ≥ 5 indicates the presence of multicollinearity. Three models were constructed for the analyses: Model 1 adjusted for age, sex, and the Nutrition Risk Screening 2002 (NRS); Model 2 adjusted for COPD, DM, CCI, and CVD; Model 3 included adjustments for creatinine, triglyceride, ALT, and GLU levels.

We employed a restricted cubic spline model to create smooth curves to examine the potential non-linear dose–response relationships between mGNRI, GNRI, NRI, and sarcopenia. In this model, the mGNRI, GNRI, and NRI were treated as continuous variables with three knots (10th, 55th, and 75th), as recommended by Harrell. Non-linearity was tested using a likelihood ratio test that compared the model with only a linear term with the model with both linear and cubic spline terms. Based on the smoothed curve, we developed a two-piecewise linear regression model to identify the threshold effect after adjusting for potential confounders. Subgroup analyses were conducted based on the subgroup variables, and heterogeneity across subgroups was assessed by adding an interaction term to the model in which the two predictor variables were multiplied.

Diagnostic value analyses were performed using ROC curves. The AUC, calculated using the C-statistic, was used to quantify the predictive ability of the logistic model for sarcopenia. The AUC between the models was compared using DeLong’s test.

All analyses were performed using R Statistical Software (Version 4.2.2^1^, The R Foundation) and Free Statistics analysis platform (Version 2.1, Beijing, China)^2^. A two-sided *p* value < 0.05 was considered statistically significant.

## 3 Results

### 3.1 Basic patient characteristics

A total of 153 patients were recruited for the study after rigorous screening based on the established inclusion and exclusion criteria. The overall prevalence of sarcopenia was 24.2% (*n* = 37; total = 153). Baseline characteristics of the groups categorized according to the presence of sarcopenia are shown in Table 2. Participants diagnosed with sarcopenia were generally older, had a higher proportion of males, and demonstrated significantly lower body weight, BMI, and nutritional indices (including GNRI, mGNRI, and NRI) than those without sarcopenia (all *p* < 0.05). Furthermore, patients with sarcopenia exhibited a greater prevalence of nutritional risk (NRS ≥ 3) and COPD, alongside lower serum albumin levels, higher total bilirubin, elevated serum creatinine, reduced triglycerides, increased C-reactive protein, and lower eGFR (all *p* values < 0.05). However, no significant differences were noted between the two groups in terms of height, body fat percentage, smoking history, alcohol consumption, diabetes mellitus, cardiovascular diseases, Charlson Comorbidity Index, hemoglobin, alanine aminotransferase, aspartate aminotransferase, urea, serum calcium, and 25-hydroxyvitamin D levels (all *p* > 0.05).

**Table 2:** Comparison of baseline characteristics between patients with and without sarcopenia.

Variables	Total (*n* = 153)	Patients without Sarcopenia (*n* = 116)	Patients with Sarcopenia (*n* = 37)	*p*
Demographic				
Sex, *n* (%)				< 0.001
Male	73 (47.7)	43 (37.1)	30 (81.1)	
Female	80 (52.3)	73 (62.9)	7 (18.9)	
Age (years), Mean± SD	80.2 ± 9.1	78.4 ± 9.1	85.6 ± 6.8	< 0.001
Anthropometry				
Height (cm)	156.5 ± 9.7	156.0 ± 9.7	158.2 ± 9.5	0.225
Weight (kg)	56.8 ± 10.9	58.1 ± 10.9	52.8 ± 9.9	0.011
BMI (kg/m2), Mean±SD	23.1 ± 3.5	23.8 ± 3.4	20.9 ± 2.7	< 0.001
BFP (%), Mean ± SD	30.1 ± 7.4	30.6 ± 7.6	28.5 ± 6.5	0.128
Life habits				
Smoking history, *n* (%)				0.578
Yes	20 (13.2)	14 (12.2)	6 (16.2)	
Drinking history, *n* (%)				0.166
Yes	12 (7.9)	7 (6.1)	5 (13.5)	
Nutritional indices				
GNRI, Mean ± SD	99.5 ± 11.4	101.7 ± 10.8	92.7 ± 10.4	< 0.001
mGRNI, Mean ± SD	54.7 ± 13.0	56.8 ± 12.8	48.1 ± 11.3	< 0.001
NRI, Mean ± SD	100.6 ± 11.5	102.8 ± 11.0	93.7 ± 10.6	< 0.001
NRS, *n* (%)				0.047
<3	75 (49.3)	62 (53.9)	13 (35.1)	
≥3	77 (50.7)	53 (46.1)	24 (64.9)	
Comorbidities				
DM, *n* (%)				0.894
Yes	51 (33.3)	39 (33.6)	12 (32.4)	
CVD, *n* (%)				0.229
Yes	42 (27.5)	29 (25)	13 (35.1)	
COPD, *n* (%)				< 0.001
Yes	24 (15.7)	10 (8.6)	14 (37.8)	
CCI, *n* (%)				0.124
Yes	66 (43.1)	46 (39.7)	20 (54.1)	
Laboratory values				
HGB (g/L), Mean ± SD	120.2 ± 23.2	121.3 ± 25.0	116.7 ± 16.4	0.298
ALT (U/L), Mean ± SD	15.0 (11.0, 21.0)	15.0 (11.0, 21.0)	14.0 (11.0, 23.0)	0.846
AST (U/L), Mean ± SD	21.9 ± 8.8	21.6 ± 8.5	23.1 ± 9.7	0.37
ALB (g/L), Mean ± SD	37.4 ± 5.5	38.0 ± 5.5	35.5 ± 5.1	0.016
TBIL (umol/L), Mean ± SD	15.1 ± 7.5	14.3 ± 5.2	17.8 ± 11.8	0.011
UREA (mmol/L), Median (IQR)	6.1 (5.0, 8.2)	6.0 (5.0, 7.7)	7.0 (4.9, 9.2)	0.446
Cr (mg/dL), Mean ± SD	1.0 ± 0.3	0.9 ± 0.3	1.1 ± 0.3	0.014
TG (mg/dL), Median (IQR)	95.6 (66.8, 138.9)	98.2 (75.9, 148.9)	81.4 (57.1, 118.6)	0.023
CRP (mg/L), Median (IQR)	2.2 (1.2, 4.2)	1.8 (1.1, 4.1)	2.8 (1.7, 6.6)	0.037
eGFR. Mean ± SD	91.9 ± 30.9	95.5 ± 31.8	80.8 ± 25.1	0.011
Serum Ca (mmol/L), Mean ± SD	2.3 ± 0.5	2.3 ± 0.6	2.2 ± 0.2	0.329
25(OH)D (ng/mL), Mean ± SD	45.9 ± 21.7	46.0 ± 22.1	45.4 ± 20.5	0.879

BMI, body mass index; BFP, body fat present, GNRI, geriatric nutrition risk index; mGNRI, modified geriatric nutrition risk index; NRI, nutritional risk index; NRS, Nutrition Risk Screening 2002; DM, diabetes mellitus; CVD, cardiovascular disease; COPD, Chronic obstructive pulmonary disease; CCI, Cavitary Cerebral Infarction; HGB, hemoglobin, ALT, Alanine Aminotransferase; AST, Aspartate Aminotransferase; ALB, Albumin, TBIL, Total Bilirubin; Cr, Creatinine; TG, triglycerides; CRP, C-reactive protein; eGFR, the estimated glomerular filtration rate.

### 3.2 Associations between nutritional indices and sarcopenia

**Figure 2:**
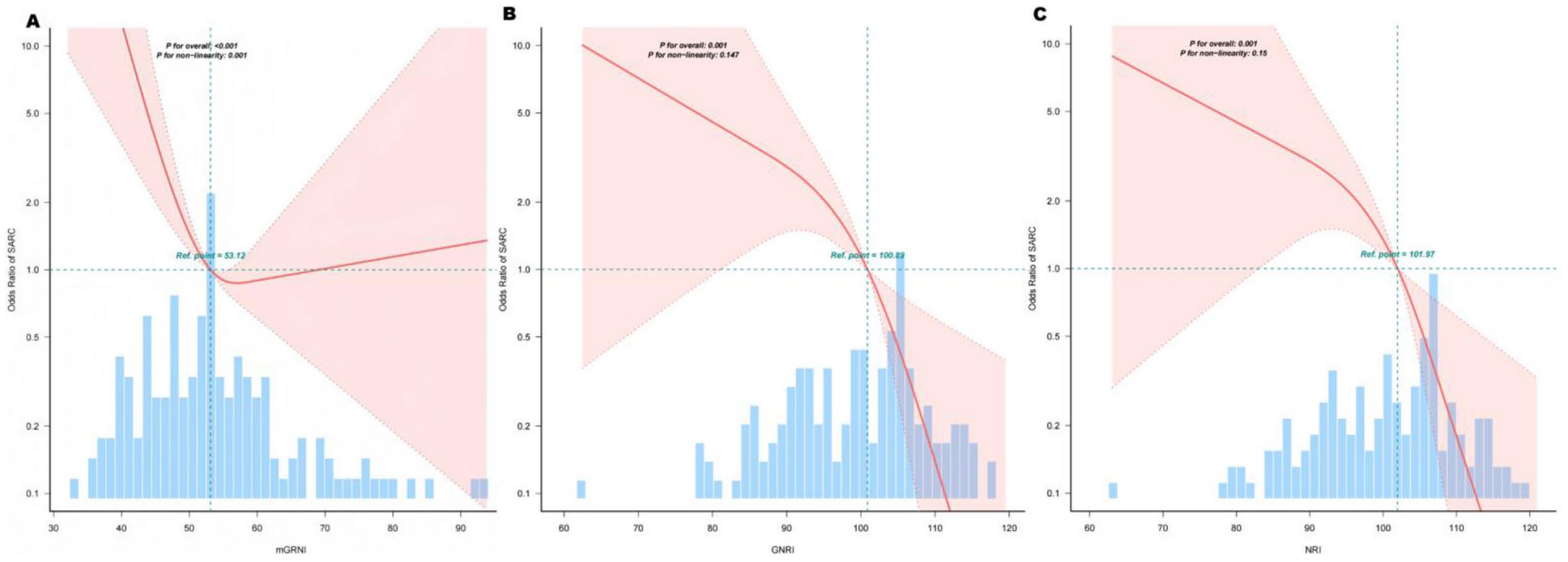
Association between mGNRI, GNRI, NRI, and sarcopenia. The solid lines represent the multivariate-adjusted hazard ratios, and the dashed lines indicate the 95% confidence intervals (CIs) derived from the restricted cubic spline regression. The knots were positioned at the 10th, 55th, and 75th percentiles of the mGNRI, GNRI, and NRI, with the highest and lowest 0.5% of each measurement trimmed. The horizontal dotted lines correspond to an odds ratio of 1.0, which serves as a reference point. Reference points were established at median GNRI and NRI levels. The regression model was adjusted for confounding variables, including age, sex, and NRS score. **(A)** Curve-fitting for mGNRI, **(B)** Curve-fitting for GNRI, **(C)** Curve-fitting for NRI.

Restricted cubic spline analyses revealed a significant L-shaped relationship between mGNRI and sarcopenia risk (*p* for non-linearity < 0.05; Figure 2A). Below the inflection point (55.48), each unit increase in mGNRI reduced sarcopenia risk by 16.8% (OR = 0.832, 95% CI: 0.741–0.934) in multivariable-adjusted models. Above 55.48, mGNRI showed no significant association with sarcopenia (*p* > 0.05), with the dose–response curve flattening along the null effect line (OR = 1.0) (Table 3). Through 1,000 bootstrap resampling validations, the 95% confidence interval for this inflection point ranges from 55.07 to 55.89, indicating that the estimate demonstrates good stability.

**Table 3:** Threshold effect analysis of the relationship of mGNRI with sarcopenia.

Inflection point	OR (95% CI)	*p* value
<55.483	0.832 (0.741–0.934)	0.0018
>55.483	1.028 (0.949–1.113)	0.5042
Likelihood Ratio test	-	0.004

The GNRI and NRI demonstrated linear protection. GNRI and NRI showed monotonic negative linear relationships with sarcopenia (*p* for non-linearity = 0.201; Figures 2B,C). Per unit increment in GNRI/NRI continuously reduced sarcopenia risk by 9% in fully adjusted models (Model III: GNRI OR = 0.91, 95% CI: 0.87–0.96; NRI OR = 0.91, 95% CI: 0.87–0.96). No critical threshold was observed; however, clinically relevant cutoffs (<98 for GNRI, <99 for NRI) identified high-risk groups with 6.93-fold elevated risk (95% CI: 2.57–18.69).

### 3.3 Multivariate logistic regression analysis

Logistic regression analyses demonstrated significant inverse relationships between all nutritional indices (mGNRI, GNRI, and NRI) and sarcopenia occurrence in both univariate and multivariate models. The results are summarized in Table 4.

**Table 4:** Logistic regression analysis, both univariate and multivariate, examining the relationships between the relevant indices and the occurrence of sarcopenia.

Variables	Non-adjusted Model OR (95%Cl)	Model I OR (95% Cl)	Model II OR (95% Cl)	Model III OR (95% Cl)
mGNRI				
	0.92 (0.88 ~ 0.96)	0.91 (0.86 ~ 0.96)	0.91 (0.87 ~ 0.96)	0.91 (0.86 ~ 0.96)
>55	1 (Ref)	1 (Ref)	1 (Ref)	1 (Ref)
<55	5.77 (2.1 ~ 15.85)	9.5 (2.74 ~ 32.96)	6.08 (2.03 ~ 18.22)	8.4 (2.69 ~ 26.2)
GNRI				
	0.93 (0.9 ~ 0.97)	0.91 (0.86 ~ 0.95)	0.93 (0.89 ~ 0.97)	0.91 (0.87 ~ 0.96)
>98	1 (Ref)	1 (Ref)	1 (Ref)	1 (Ref)
<98	5.25 (2.34 ~ 11.78)	7.72 (2.73 ~ 21.82)	5.64 (2.31 ~ 13.76)	6.93 (2.57 ~ 18.69)
NRI				
	0.93 (0.9 ~ 0.97)	0.91 (0.87 ~ 0.95)	0.93 (0.89 ~ 0.97)	0.91 (0.87 ~ 0.96)
>99	1 (Ref)	1 (Ref)	1 (Ref)	1 (Ref)
<99	5.25 (2.34 ~ 11.78)	8.02 (2.74 ~ 23.52)	5.64 (2.31 ~ 13.76)	6.93 (2.57 ~ 18.69)

Model I: Adjustment for AGE, SEX, and NRS.

Model II: Adjustment for COPD, DM, CVD, and CCI.

Model III: Adjustment for ALT, Cr, GLU, and TG.

GNRI, geriatric nutrition risk index; mGNRI, modified geriatric nutrition risk index; NRI, nutritional risk index.

Each unit increase in the mGNRI was associated with a 9% reduction in sarcopenia risk in the fully adjusted Model III (OR = 0.91, 95% CI: 0.86–0.96). This protective effect remained consistent across all adjustment models. Similar trends were observed for GNRI (Model III OR = 0.91, 95% CI: 0.87–0.96) and NRI (Model III OR = 0.91, 95% CI: 0.87–0.96), indicating comparable protective effects per unit increment.

Subjects with suboptimal nutritional status exhibited substantially elevated sarcopenia risk:mGNRI <55: Adjusted OR = 8.40 (95% CI: 2.69–26.20) in Model III, representing an 8.4-fold increased risk versus the reference group (mGNRI >55). GNRI <98: Adjusted OR = 6.93 (95% CI: 2.57–18.69) in Model III. NRI < 99: Adjusted OR = 6.93 (95% CI: 2.57–18.69) in Model III. The associations persisted after sequential adjustment for covariates.

### 3.4 Subgroup analyses

**Figure 3:**
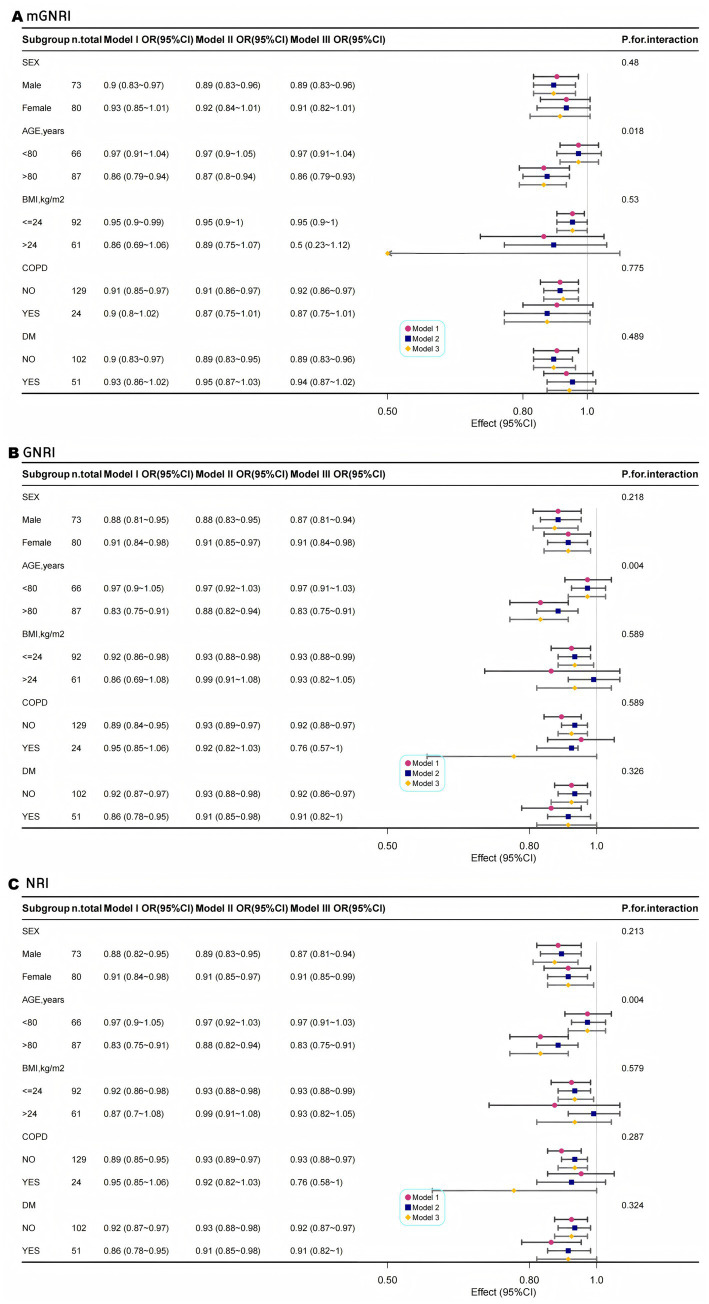
Multimodal subgroup logistic regression analysis and forest plots of the association between mGNRI **(A)**, GNRI **(B)**, NRI **(C)**, and sarcopenia.

Multimodal subgroup analyses were performed for age, sex, BMI, COPD, and DM; consistent with our hypotheses, our findings indicated that the associations between mGNRI, GNRI, NRI, and sarcopenia were statistically significant across all subgroups. Although the interaction *p*-values for age and all nutritional indices (mGNRI, GNRI, and NRI) were found to be less than 0.05, the clinical significance of these results may be limited due to the presence of multiple testing and the consistent directionality of the associations observed. Conversely, a consistent risk reduction was evident in younger adults, males, and patients without COPD, supporting age-targeted nutritional interventions (Figures 3A–C).

### 3.5 Comparison of the diagnostic efficacy of nutritional indices for sarcopenia

**Figure 4:**
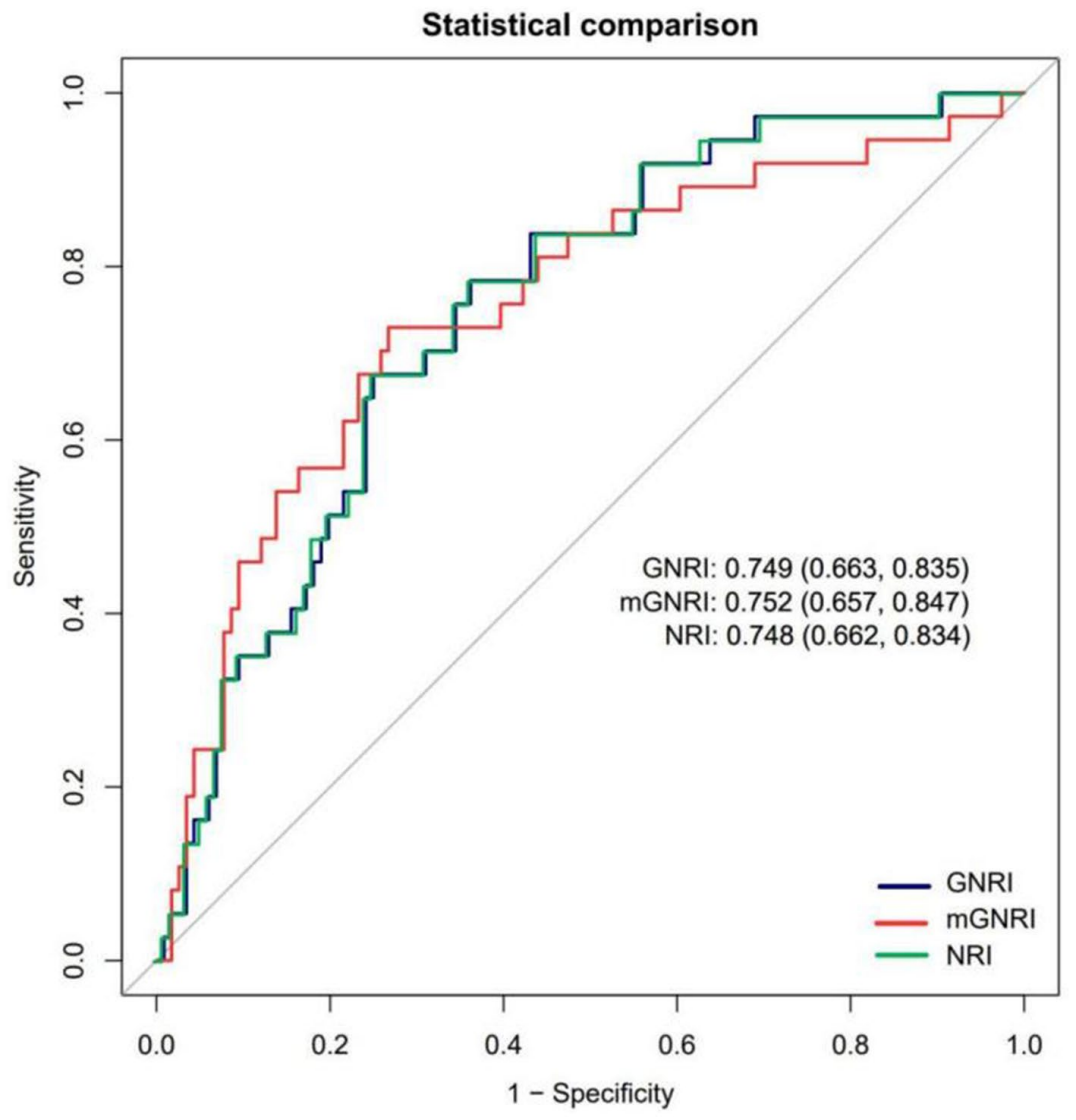
Receiver operating characteristic curves assessing the predictive ability of mGNRI, GNRI, and NRI.

The analysis of the ROC curve indicated that all three nutritional indices exhibited moderate and comparable diagnostic performance for sarcopenia, with no statistically significant differences in their AUCs (mGNRI: 75.21% [65.68–84.74]; GNRI: 74.91% [66.33–83.48]; NRI: 74.81% [66.22–83.40]; all pairwise *p* > 0.05 by DeLong’s test), as illustrated in Figure 4. Notably, the mGNRI showed a sensitivity exceeding 80% at a cutoff value of 55. This finding aligns closely with the previously established L-shaped risk inflection point of 55.48, reinforcing its potential as a screening tool for high-risk populations. Furthermore, the standard cutoff values for GNRI and NRI of less than 98 and 99, respectively, offer a more balanced sensitivity and specificity of approximately 75 and 60%, respectively, making them appropriate for clinical diagnostic support. The mGNRI demonstrated the highest sensitivity (72.97%), indicating its suitability as a screening tool for maximizing the identification of potential sarcopenia patients within the population (reducing missed diagnoses). The relevant sensitivity, specificity, optimal cutoff values, AUC, Youden index critical values, and pre-test/post-test probability scenarios for mGNRI, GNRI, and NRI can be found in the Supplementary materials.

## 4 Discussion

This study provides a comprehensive evaluation of the association patterns and diagnostic effectiveness of three nutritional-inflammatory composite indices (mGNRI, GNRI, and NRI) in relation to sarcopenia among hospitalized older patients. This population-based retrospective cross-sectional study revealed that these indices (mGNRI, GNRI, and NRI) are independently associated with a reduced risk of sarcopenia. Our principal findings indicate that the mGNRI demonstrates an L-shaped nonlinear relationship with sarcopenia, with a threshold identified at 55.48; specifically, for each unit increase below this threshold, the risk of sarcopenia decreased by 16.8% (OR = 0.832). In contrast, both the GNRI and NRI exhibited a negative linear correlation, with each unit increase corresponding to a 9% reduction in risk (OR = 0.91). All indices significantly predicted a high risk of sarcopenia at their respective critical values (mGNRI < 55, GNRI < 98, and NRI < 99), with odds ratios ranging from 6.93–8.40. Furthermore, the mGNRI showed optimal diagnostic sensitivity (>80% at a cutoff of 55), whereas the GNRI and NRI provided a more balanced sensitivity/specificity ratio of approximately 75%/60%. These findings have important implications for the management of sarcopenia, particularly in East Asian populations. Meanwhile, it is important to emphasize that patients with baseline acute inflammation (defined as CRP > 10 mg/L or with relevant clinical diagnoses) were excluded during the design phase of this study. This means that the primary associative results we report have inherently controlled for the potential confounding effects of acute inflammatory status, further enhancing the robustness of our findings applicable to elderly hospitalized patients.

Previous studies have extensively examined the relationship between the GNRI and sarcopenia. Specifically, Hao et al. demonstrated that the GNRI serves as a dependable predictor of sarcopenia in individuals aged ≥ 45 years in the United States. Their findings revealed a significantly lower prevalence of sarcopenia among those with a high GNRI, establishing a nonlinear inverse correlation at a GNRI threshold of 91.935 (19). Duan et al. indicated that a low GNRI correlated with an elevated risk of osteosarcopenia in older adults diagnosed with type 2 diabetes mellitus (T2DM). Utilizing a straightforward tool, such as the GNRI, for a thorough clinical assessment of nutritional status could facilitate the early detection of individuals at an increased risk of osteosarcopenia among older diabetic patients (20). To evaluate the effectiveness of the GNRI in identifying frailty and sarcopenia in hospitalized older patients, El-Kawaly et al. conducted a study of 155 hospitalized individuals and concluded that the GNRI was an effective and simple method for screening both sarcopenia and frailty (21). Recent investigations into the mGNRI have predominantly focused on oncology (17, 22). A multicenter cohort study examined the efficacy of integrating the modified mGNRI with handgrip strength measurements to assess cancer prognosis. These findings indicate that this combined approach offers robust prognostic stratification for patients with cancer, as well as the ability to predict physical frailty, malnutrition, and cachexia (22).

Furthermore, the relationship between sarcopenia and inflammatory factors has always been a focus of research. Liu et al. systematically evaluated various biomarkers associated with sarcopenia and noted that inflammatory biomarkers are the most extensively studied category, including IL-6, CRP, and TNF-*α*. These inflammatory factors are generally elevated in patients with sarcopenia, and their expression levels decrease following intervention, suggesting that inflammation plays a significant role in the pathogenesis of sarcopenia (23). In addition, a large meta-analysis by Tuttle et al. (24) established a significant negative correlation between systemic inflammatory markers (e.g., CRP, IL-6, TNF-α) and both muscle strength and mass across diverse adult populations (24). In future studies, we need to detect multiple indicators simultaneously to build a more comprehensive spectrum of inflammation.

In our study, we observed an L-shaped correlation between mGNRI and sarcopenia. This suggests that the impact of the mGNRI may encounter a “saturation phenomenon”; once the nutritional-inflammatory status exceeds a specific threshold (mGNRI > 55.48), the additional benefits of further optimization to prevent sarcopenia become negligible. This phenomenon can be attributed to several factors. First, CRP serves as an indicator of the baseline inflammatory status at lower levels, and its reduction can significantly improve muscle metabolism. However, when CRP falls below 1 mg/L (corresponding to mGNRI > 55.48), it enters a physiological fluctuation range, and further reductions have minimal impact on the muscle (25). Second, the ratio of actual weight to ideal weight used in mGNRI may be exaggerated in obese individuals, masking the true extent of muscle loss (referred to as “obesity-related sarcopenia”), thereby weakening the correlation within the higher mGNRI range.

In clinical practice, patients with a modified mGNRI of less than 55 should receive enhanced anti-inflammatory treatment along with high-protein nutritional support. For individuals with a GNRI < 98 or NRI < 99, we recommend standard protein supplementation in conjunction with resistance training. For patients whose mGNRI falls between 55 and 60, it is advisable to re-evaluate CRP levels and weight every 3 months to mitigate the risk of inflammation recurrence. Consequently, the mGNRI can be prioritized in clinical screening due to its high sensitivity (greater than 80%) and low miss rate. It is advisable for clinicians to integrate the mGNRI into the Comprehensive Geriatric Assessment (CGA) and to implement combined anti-inflammatory and nutritional strategies for patients presenting with low mGNRI values (mGNRI < 55).

However, this study had some limitations. First, as a cross-sectional study, it does not allow for causal inferences; therefore, future prospective research is necessary to confirm the effects of mGNRI dynamics on sarcopenia progression. Second, a potential selection bias exists because of the use of a single-center inpatient cohort, which may limit the applicability of the findings to broader community populations (for instance, the reported 15.7% prevalence of COPD may not be representative of wider demographics). Another limitation of this study is the relatively limited number of sarcopenia events. Although we conservatively followed methodological literature recommendations by using a three-knot restricted cubic spline for exploratory analysis, the sample size remains suboptimal, posing a risk of overfitting. Finally, in the clinical database utilized for this study, the levels of IL-6 and TNF-*α* were not systematically collected and measured for all participants. Therefore, this study only analyzed CRP and did not include other key inflammatory factors such as IL-6 and TNF-α, which represents a significant limitation.

This study validated that the mGNRI, which incorporates inflammation (as indicated by CRP levels) and nutritional reserves (body weight relative to ideal body weight), exhibits high sensitivity (> 80%) for identifying sarcopenia in hospitalized older patients. The correlation with L-type suggests a critical “golden window” for intervention, indicating that the most significant benefits arise when mGNRI falls below 55. Both the GNRI and NRI function as linear metrics that are appropriate for the ongoing assessment of nutritional status.

## Data Availability

The raw data supporting the conclusions of this article will be made available by the authors, without undue reservation.

## Footnotes

^1^ http://www.R-project.org

^2^ http://www.clinicalscientists.cn/freestatistics
