# Supplementary material for: Modified geriatric nutritional risk index: a high-sensitivity marker with L-shaped association for sarcopenia in hospitalized older adults
Source: Front Nutr. 2025 Nov 4;12:1686537. doi: 10.3389/fnut.2025.1686537 (PMC12623325; doi:10.3389/fnut.2025.1686537)
Supplement: Supplementary file 1 [file Data_Sheet_1.PDF]

Supplementary Materials

TABLE 1Threshold effect analysis

Item	Breakpoint.OR (95%CI)	P value
E_BK1	55.483 (55.073 ,55.893)	
slope1	0.832 (0.741~0.934)	0.0018
slope2	1.028 (0.949~1.113)	0.5042
Likelihood Ratio test	-	0.004

TABLE 2 The Diagnostic Value of Different Nutritional Risk Indices for Sarcopenia: ROC Analysis Results

	optimal cutoff values	AUC (95% CI)	sensitivity(95%CI)	specificity(95%CI)	Youden index
mGNRI	0.276	75.21% (65.68% - 84.74%)	72.97%	73.28%	1.463
GNRI	0.261	74.91% (66.33% - 83.48%)	67.57%	75.00%	1.426
NRI	0.263	74.81% (66.22% - 83.40%)	67.57%	75.00%	1.426

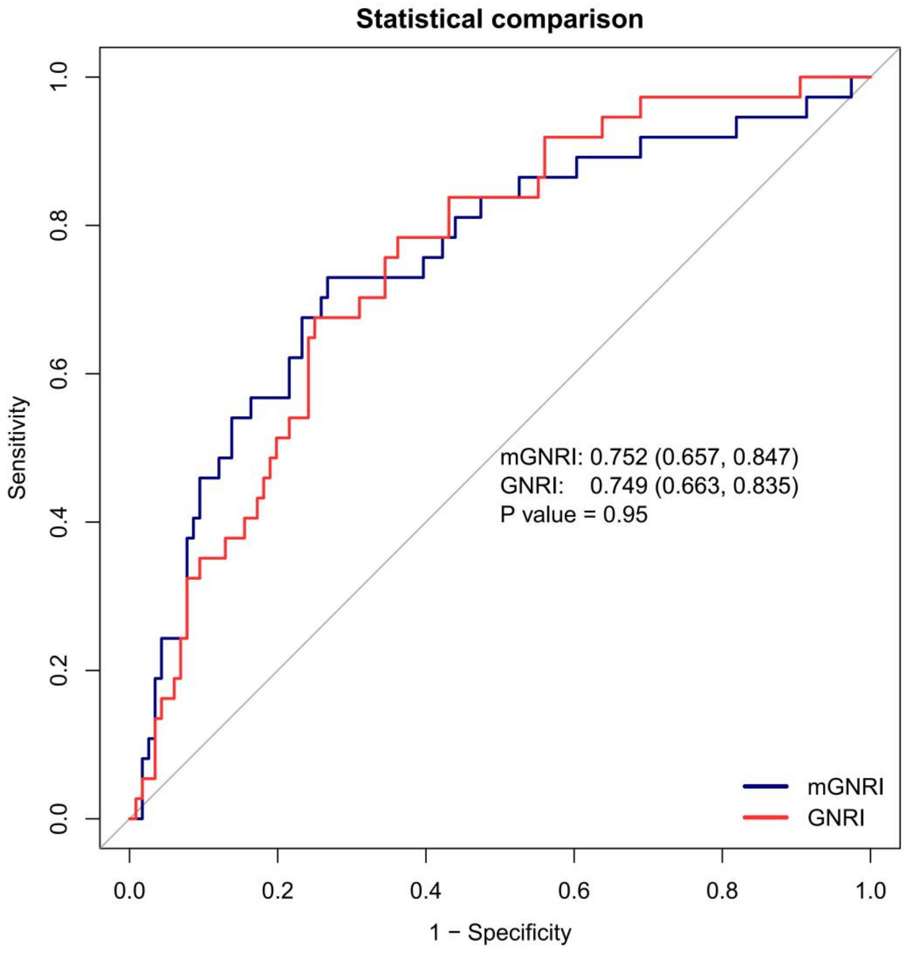
Statistical comparison
mGNRI: 0.752 (0.657, 0.847)
GNRI: 0.749 (0.663, 0.835)
P value = 0.95
mGNRI
GNRI
Sensitivity
1 − Specificity
0.0
0.2
0.4
0.6
0.8
1.0

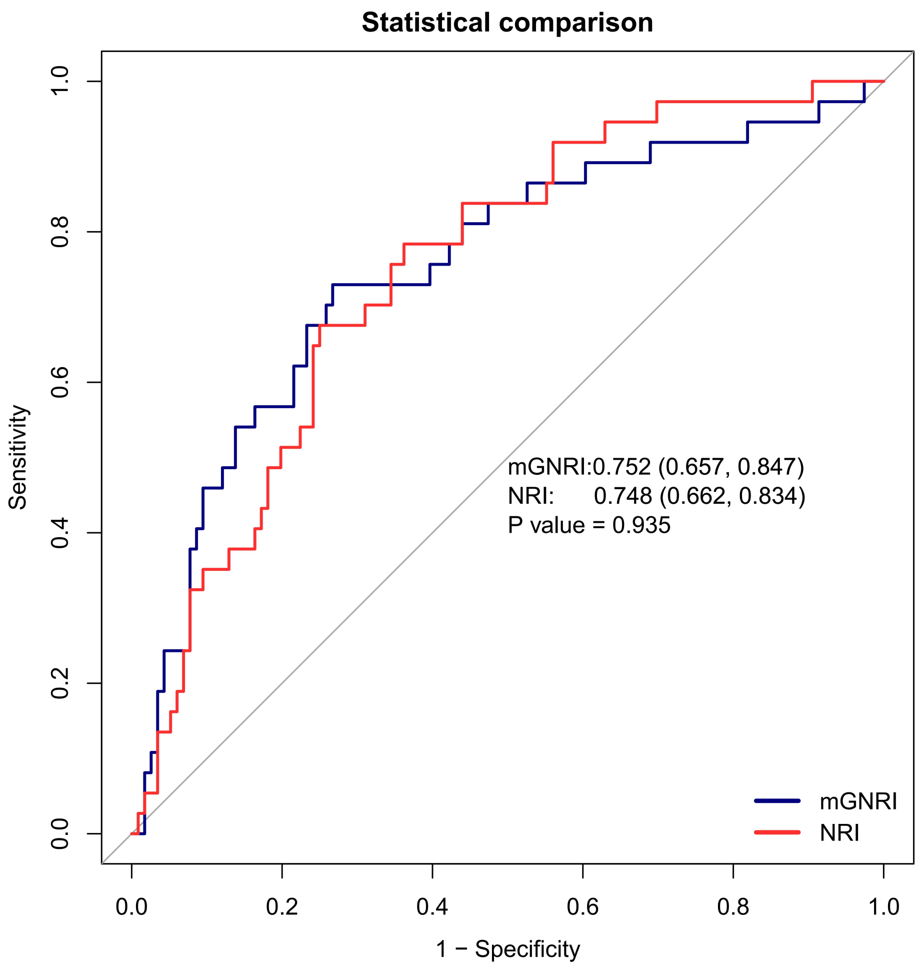

Statistical comparison
Sensitivity
1 − Specificity
0.0
0.2
0.4
0.6
0.8
1.0
mGNRI:0.752 (0.657, 0.847)
NRI: 0.748 (0.662, 0.834)
P value = 0.935
mGNRI
NRI

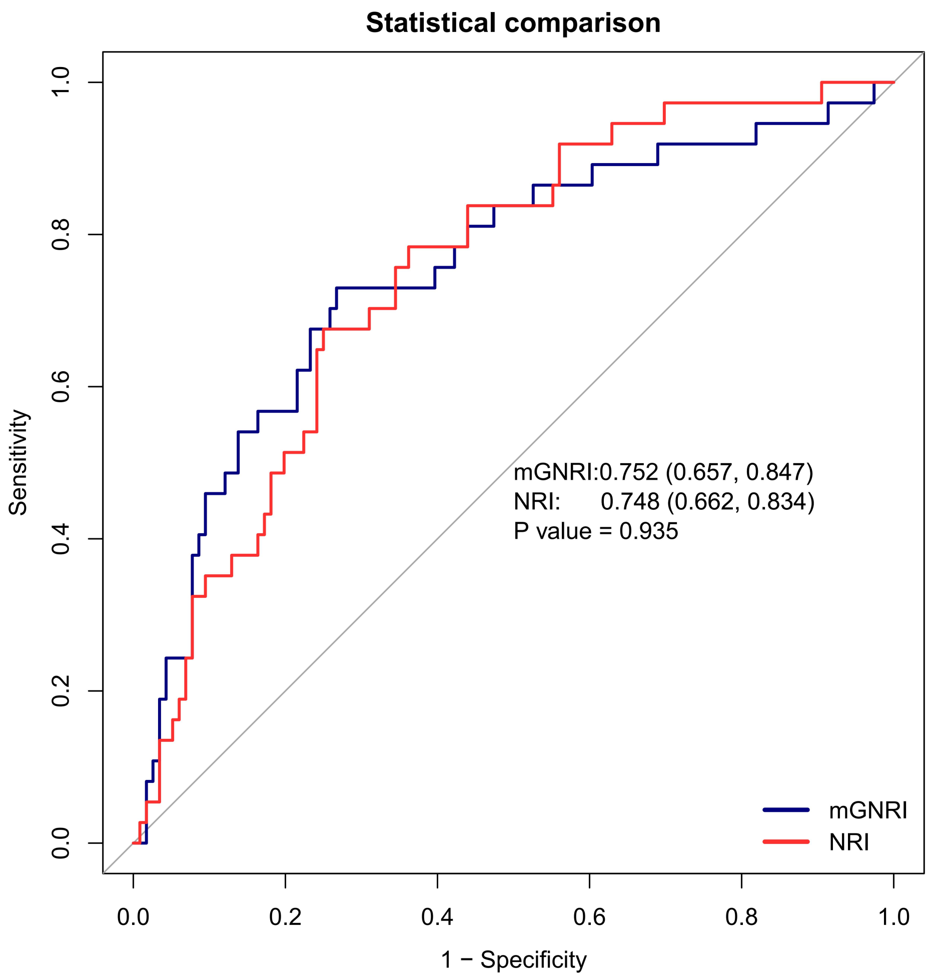

TABLE 3 Diagnostic Performance of mGNRI for Sarcopenia (Cut-off = 55)

Metric	Definition / Calculation	Value in Cohort (95% CI)
Pre-test Probability (Prevalence)	(Patients with Sarcopenia / Total Patients) × 100%	24.2%
Sensitivity	(True Positives / All Patients with Sarcopenia) × 100%	72.97%
Specificity	(True Negatives / All Patients without Sarcopenia) × 100%	73.28%
Positive Predictive Value (PPV)	Probability of having sarcopenia given a positive test	45.2%
Negative Predictive Value (NPV)	Probability of being free of sarcopenia given a negative test	90.1%
